# Network analysis of organ donation willingness, attitude, and death among Chinese university students

**DOI:** 10.3389/fpubh.2026.1783464

**Published:** 2026-05-25

**Authors:** Lele Xiao, Zheng Ren, Haoyang Li

**Affiliations:** 1 School of Politics and Public Administration, Guangxi Normal University, Guilin, China; 2 Affiliated Hospital of Xuzhou Medical University, Xuzhou, Jiangsu, China; 3 Jiangsu Provincial Organ Procurement Organization, Nanjing, Jiangsu, China; 4 Western Urban and Rural Integration Development Institute, Guilin, China

**Keywords:** death attitudes, donation willingness, network analysis, organ donation, university students

## Abstract

**Purpose:**

The psychological mechanisms underlying university students’ willingness to donate, particularly the interplay among donation attitudes, death attitudes, and donation willingness, remain insufficiently understood. Conventional regression approaches are limited in capturing the complex interrelationships among these psychological constructs, highlighting the need for a more integrative analytical framework.

**Methods:**

A cross-sectional survey was conducted among 960 Chinese university students from December 23 to 31, 2024. The questionnaire included organ donation attitude scale, the Death Attitude Profile-Revised, and demographic information. Network analysis was performed using the EBICglasso algorithm with LASSO regularization to estimate a Gaussian graphical model. Node strength and bridge strength were computed as centrality indices, with stability assessed via 1,000 bootstrap samples.

**Results:**

Network analysis identified reasons for obstructing organ donation as the most central node (strength = 2.07), followed by approach acceptance (strength = 0.66) and disagreement with the value of organ donation (strength = 0.65). Bridge centrality analysis further revealed that reasons for obstructing organ donation (bridge strength = 1.59) and donation willingness (bridge strength = 1.05) served as key connectors linking the donation attitude and death attitude communities. Stability analyses demonstrated robust centrality estimates (CS-coefficient = 0.62 for node strength and 0.75 for bridge strength).

**Conclusion:**

The findings suggest that perceived barriers to donation, positive valuation of donation, and adaptive death attitudes play pivotal roles within the psychological network influencing donation willingness. To enhance organ donation rates, interventions should simultaneously address cognitive barriers, incorporate death education, and strengthen value-based advocacy. These culturally tailored strategies may provide a foundation for promoting voluntary organ donation among university students in China.

## 1 Introduction

Organ transplantation has revolutionized modern medicine by saving countless lives of patients with end-stage organ failure (1). However, the persistent shortage of transplantable organs remains a critical global health challenge. In China, despite the establishment of a national organ donation system in 2015, the donation rate remains low at only 3-4 donors per million population, far below that of countries such as Spain (49.6 per million) and the United States (36 per million) (2). Since China banned the use of organs from executed prisoners in 2015, voluntary citizen donation has become the sole legal source of transplantable organs (3). This policy shift represents a major reform in China’s organ transplantation system, aligning it with international ethical standards. Nevertheless, this transition poses substantial challenges, as the voluntary donation rate remains insufficient to meet clinical demand. As a result, more than 300,000 patients in China await organ transplantation each year, yet fewer than 10% ultimately receive a transplant. Enhancing citizens’ willingness to donate organs has become a key bottleneck in advancing organ transplantation in China (4, 5).

The Knowledge-Attitude-Practice (KAP) framework is a widely adopted model in health education and behavioral intervention research, used to understand and modify individual behaviors (6). In the field of organ donation, the KAP framework has been widely applied to assess knowledge, attitudes, and intentions toward organ donation among the general public, healthcare professionals, and specific populations such as medical students (6–8). Within this framework, knowledge refers to an individual’s awareness and understanding of organ donation–related information, including relevant policies, basic medical concepts, and procedural standards (6). Previous studies have shown that the general public has a relatively low level of knowledge about organ donation. Insufficient knowledge is considered a major barrier to the formation of positive attitudes and donation intentions (8, 9). Attitude refers to an individual’s beliefs, feelings, and evaluations regarding organ donation (9). A positive attitude is typically characterized by approval, support, and endorsement of organ donation (6, 10). In the context of organ donation, because donation typically occurs after death or under specific medical conditions, “practice” is often operationalized as donation willingness or registration as an organ donor (8). Research has shown that higher levels of knowledge and more positive attitudes significantly predict donation willingness (7, 8, 11). Empirical evidence from a large-scale study involving 4,274 participants demonstrated a positive association between favorable attitudes toward organ donation and increased willingness to donate among the Chinese general population (7). Given this association, it is essential to further explore and enhance public attitudes toward organ donation.

The intrinsic nature of organ donation, occurring only after the determination of death, either brain or cardiac death, creates an inherent link to the concept of mortality. Prior to Templer’s seminal work on death anxiety (12), discussions of death were largely neglected in the field of mental health. Because death is universally associated with pain and existential cessation, most individuals tend to respond negatively, often experiencing feelings of dread and avoidance (13, 14). Nevertheless, contemporary psychological research suggests that attitudes toward mortality exist along a continuum encompassing both negative and adaptive responses (15–17). Wong and colleagues categorized attitudes toward death into two dimensions, positive and negative, thereby expanding the conceptual understanding of mortality (18). These dimensions are conceptually and functionally distinct, and therefore may exhibit differential associations with organ donation attitudes and willingness. Fear of death and death avoidance, as negative and defensive orientations, are hypothesized to inhibit donation willingness by activating mortality-related threat and subsequent avoidance responses. In contrast, approach acceptance, which involves a proactive embrace of death as a meaningful transition, may facilitate altruistic end-of-life behaviors such as organ donation by reframing death as an opportunity for legacy building. Escape acceptance, while positive in valence, reflects a desire to escape suffering rather than an active prosocial motive; thus its association with donation willingness may be weaker or indirect. Neutral acceptance, characterized by a balanced and calm acknowledgment of death, may show minimal or inconsistent associations due to its emotionally detached nature. These theoretical distinctions provide a conceptual basis for examining how specific death attitude dimensions uniquely relate to organ donation attitudes and willingness, moving beyond a simple positive–negative dichotomy. A study involving 478 Chinese college students found that positive and negative death attitudes exert distinct influences on willingness to donate one’s body (19). These findings suggest a potentially important yet underexplored association between organ donation willingness and attitudes toward death. However, this relationship remains insufficiently explored in the existing literature.

Numerous studies have examined factors influencing individuals’ willingness to donate organs as well as the organ donation process. A systematic review highlighted that specific aspects of family conversations, particularly their timing and context, can substantially influence relatives’ decisions regarding organ donation (20). Encouragement by healthcare professionals to engage families in discussions about organ donation has been shown to improve donation rates (21). In China, extensive research has been conducted on factors influencing organ donation willingness and attitudes. Huang et al. (22) reported that families with smaller or simpler structures were more likely to reach consensus on organ donation decisions. A nationwide survey involving 11,301 participants found that social support and health literacy significantly influence attitudes toward organ donation (23). Furthermore, studies focusing on young adults indicate that although most individuals are aware of organ donation, their understanding remains superficial (24).

As a key group in social transformation, university students demonstrate greater openness to new ideas, making them a critical population for promoting organ donation initiatives in China. Enhancing university students’ knowledge and willingness regarding organ donation has important implications for advancing China’s organ transplantation system. A critical step is to accurately identify the key determinants influencing students’ willingness to donate organs. Most existing studies rely on linear regression models, which are limited in their ability to capture complex interrelationships among variables. Although university students are not the primary population for immediate organ donation, selecting this sample is justified on theoretical and practical grounds. Under China family consent requirement, students will serve as future decision makers for deceased relatives, a role that directly influences donation rates from older adults. Attitudes formed during young adulthood also demonstrate relative stability and predict donation behaviors in later years. Furthermore, as a group more receptive to new ideas, students can initiate family discussions about donation and influence older adults relatives’ attitudes. The accessibility of university settings further facilitates developing and testing evidence-based interventions. Therefore, the present study employs network analysis to examine the complex interrelationships among organ donation attitudes, donation willingness, and death attitudes among Chinese university students, with the aim of identifying central nodes that may inform targeted intervention strategies. Based on the theoretical framework outlined above, we formulated specific, testable hypotheses regarding the expected network structure. First, we hypothesized that positive organ donation attitudes, particularly agreement with the value of donation (Avod), would exhibit a positive edge with donation willingness, whereas negative attitudes, specifically reasons for obstructing donation (Rood) and disagreement with the value of donation (Dvod), would show negative edges with willingness. Second, regarding death attitudes, we expected that approach acceptance (Maa), reflecting a proactive and meaningful embrace of mortality, would be positively associated with donation willingness, while fear of death (Mdf) and death avoidance (Mde) would be negatively associated with willingness. Third, we anticipated that approach acceptance would act as a bridge node connecting the death attitude cluster to the donation attitude and willingness clusters, given its theoretical role in facilitating legacy-building altruistic behaviors. These hypotheses guided our network estimation and centrality analyses.

## 2 Methods

### 2.1 Participant recruitment and data collection procedures

Prior to data collection, administrative personnel at the participating universities were contacted to obtain institutional approval and support. Four universities were included: Xuzhou Medical University, China University of Mining and Technology, and Jiangsu Normal University (all located in Xuzhou, Jiangsu Province, eastern China), as well as Guangxi Normal University (Guilin, Guangxi Province, southern China). The study has been approved by the Ethics Committee of the Affiliated Hospital of Xuzhou Medical University (Number: XYFY2024-KL522-01). After approval was obtained, an electronic questionnaire with an embedded QR code was distributed via official university channels to recruit participants.

A convenience sampling approach was adopted, and data were collected via the Wenjuanxing online survey platform over a one-week period (December 23–31, 2024). A total of 1,000 university students who were invited and agreed to participate initiated the online survey (250 per university). After applying predefined inclusion and exclusion criteria (see below), 960 valid responses were retained, resulting in a completion rate of 96.0% (960/1000).

#### 2.1.1 Inclusion criteria

(1) Voluntary participation with informed consent;
(2) Demonstrated comprehension of questionnaire items as assessed by attention-check measures. Attention-check items were designed without substantive meaning and required participants to select a predetermined response to identify inattentive or careless responses.

#### 2.1.2 Exclusion criteria

(1) Incomplete questionnaires or those with more than 10% missing responses;
(2) Response times indicative of inattention (less than 30% or greater than 200% of the median completion time);
(3) Logical inconsistencies across items (e.g., contradictory responses to comparable questions);
(4) Failure to correctly respond to embedded validity-check items;
(5) Straightlining (i.e., identical responses across all items).

### 2.2 Measurement instruments

The questionnaire comprised two main sections and incorporated additional quality control measures:

Section 1: sociodemographic characteristics.

This section collected background information, including demographic variables (e.g., gender, age, ethnicity) and socioeconomic indicators (e.g., place of residence, religious affiliation).

Section 2: standardized psychological scales.

#### 2.2.1 Organ donation attitude scale

Organ donation attitudes were measured using a validated instrument developed by See et al. (25), comprising three theoretically grounded subscales assessing distinct dimensions of organ donation attitudes. The scale includes 22 items across three dimensions: (1) reasons for obstructing organ donation (item 9 and items 12–22), (2) agreement with the value of organ donation (items 1–6), and (3) disagreement with the value of organ donation (items 7-8 and 10-11). Participants rated each item on a 7-point Likert scale ranging from 1 (“strongly disagree”) to 7 (“strongly agree”).

The scale demonstrated excellent internal consistency (Cronbach’s *α* = 0.896). Subscale reliabilities were also acceptable: agreement with the value of donation (Avod, *α* = 0.926), reasons for obstructing donation (Rood, *α* = 0.929), and disagreement with the value of donation (Dvod, *α* = 0.797). Although the scale has demonstrated structural validity in Chinese populations, confirmatory factor analysis was not conducted in the present study. Total scores were computed such that higher scores indicated more favorable attitudes toward organ donation, with negatively worded items reverse-coded.

Organ donation willingness was assessed using a single item adapted from the scale developed by See et al. (25), specifically Item 1 of the donation intention subscale. Previous studies have demonstrated that single-item measures can validly assess organ donation willingness (7, 26). Accordingly, this item was used to assess donation willingness. The item directly assessed willingness to donate organs, with five response options ranging from (1) “unwilling to donate” to (5) “willing to donate and have already signed an organ donation consent card.” Higher scores indicate greater willingness. The remaining items assess family consent, specific organs, recipients, and influential opinions, but were not included as primary indicators in the network analysis, consistent with prior studies. The single item was treated as a continuous variable.

#### 2.2.2 Death Attitude Profile-Revised (DAP-R)

The Chinese version of the Death Attitude Profile-Revised (DAP-R), originally developed by Wong et al. (18), was used to assess multidimensional attitudes toward death. This validated 32-item instrument assesses five dimensions: three positive (escape acceptance [8 items], approach acceptance [5 items], and neutral acceptance [5 items]) and two negative (fear of death [7 items] and death avoidance [7 items]). Participants rated each item on a 5-point Likert scale ranging from 1 (“strongly disagree”) to 5 (“strongly agree”). Subscale scores were calculated by summing item responses within each dimension, with higher scores indicating stronger endorsement of the corresponding attitude.

The scale demonstrated excellent internal consistency (Cronbach’s *α* = 0.938), consistent with previous validation studies among Chinese university students (27). Subscale reliabilities were as follows: fear of death (*α* = 0.879), death avoidance (*α* = 0.868), approach acceptance (*α* = 0.903), escape acceptance (*α* = 0.887), and neutral acceptance (*α* = 0.832).

#### 2.2.3 Quality control measures

Two validity-check items were embedded in the questionnaire. Each item instructed participants to select a predetermined response (e.g., “select ‘strongly agree’”). Participants who failed to select the required response on either validity-check item were excluded. Questionnaires exhibiting straightlining (i.e., identical responses across all Likert-scale items) were also excluded. These procedures were applied prior to deriving the final sample of 960 participants.

### 2.3 Ethical considerations

The research protocol was approved by the Institutional Review Board of Affiliated Hospital of Xuzhou Medical University (No. XYFY2024-KL522-01). All procedures complied with the ethical principles of the Declaration of Helsinki, including (1) voluntary participation with informed consent, (2) the right to withdraw without penalty, (3) anonymity of responses, and (4) secure data storage.

### 2.4 Data analysis

The cleaned dataset was analyzed using SPSS 26.0 and R (packages: qgraph, bootnet, and networktools). Descriptive statistics were used to summarize demographic characteristics and variable distributions. Group differences were examined using independent-samples *t*-tests or one-way ANOVA.

#### 2.4.1 Network estimation

Network analysis was conducted using the EBICglasso (Extended Bayesian Information Criterion graphical lasso) algorithm to estimate a Gaussian graphical model based on partial correlations, with LASSO regularization applied to reduce spurious edges. The tuning parameter *γ* was set to 0.5 to balance model fit and sparsity. Spearman correlations were used due to the ordinal nature of the data.

#### 2.4.2 Centrality

Node strength was used as the primary centrality index, defined as the sum of absolute edge weights connected to a node. This choice was based on the limitations of betweenness and closeness centrality in psychological networks (28). Closeness and betweenness centrality rely on assumptions of shortest paths and information flow, which may not hold in psychological networks. In contrast, strength centrality captures the total weight of direct connections and is considered more appropriate and interpretable in psychological network analysis.

#### 2.4.3 Bridge analysis

Bridge centrality was assessed using bridge strength, defined as the sum of edge weights connecting a node to nodes in other clusters. Three clusters were predefined based on theoretical considerations: (1) donation attitude (Rood, Avod, Dvod); (2) death attitude (Mna, Mea, Maa, Mde, Mdf); and (3) willingness (Willingness), treated separately to avoid circularity. Bridge strength was computed using the networktools package.

#### 2.4.4 Stability

Network stability was evaluated using 1,000 bootstrap samples implemented in the bootnet package. The correlation stability (CS) coefficient was interpreted as follows: values above 0.25 indicate moderate stability and values above 0.50 indicate high stability. The CS coefficients were 0.62 for node strength and 0.75 for bridge strength, indicating good to excellent stability.

#### 2.4.5 Visualization

Network visualization was performed using the Fruchterman–Reingold layout implemented in qgraph. Edge thickness was proportional to the absolute edge weight, with positive edges shown in blue and negative edges in red.

## 3 Results

### 3.1 Preliminary analysis

Harman’s single-factor test was conducted to assess common method bias. The first factor accounted for 25.82% of the total variance, which was below the conventional 40% threshold (29). This result indicates that common method bias was not a serious concern in the present study.

A total of 960 Chinese university students were included in the final analysis, with a mean age of 19.59 years (range: 18–23). Among the participants, 609 (63.44%) were male and 351 (36.56%) were female. City/down (*n* = 481, 50.10%) and rural (*n* = 479, 49.90%) household registrations were nearly equally represented. A total of 27 participants (2.81%) reported a religious affiliation. The mean organ donation willingness score was 2.10 ± 0.94, corresponding to the stage of “having considered but not yet decided.”

Independent-samples *t*-tests and one-way ANOVA were conducted to compare organ donation willingness across demographic subgroups. As shown in Table 1, no statistically significant differences in organ donation willingness were observed across gender (male: 2.09 ± 0.97 vs. female: 2.11 ± 0.89; *t* = −0.364, *p* = 0.716), age (*F* = 0.978, *p* = 0.398), household registration (city/down: 2.07 ± 0.94 vs. rural: 2.13 ± 0.95; *t* = −0.931, *p* = 0.352), or religious affiliation (no religion: 2.09 ± 0.93 vs. with religion: 2.30 ± 1.30; *t* = −0.817, *p* = 0.421).

**Table 1:** Comparison of scores of college students with different demographic characteristics.

Variables	Categories	*N* (%)	Donation willingness		
			Mean ± SD	*T*/*F*	*p*
Gender	Male	609 (63.44)	2.09 ± 0.97	−0.364	0.716
	Female	351 (36.56)	2.11 ± 0.89		
Age	18	291 (30.31)	2.02 ± 0.94	0.978	0.398
	19	279 (29.06)	2.14 ± 0.92		
	20	174 (18.12)	2.15 ± 0.99		
	≥21	216 (22.50)	2.09 ± 0.94		
House registration	City/town	481 (50.10)	2.07 ± 0.94	−0.931	0.352
	Rural	479 (49.90)	2.13 ± 0.95		
Religious belief	No	933 (97.19)	2.09 ± 0.93	−0.817	0.421
	Yes	27 (2.81)	2.30 ± 1.30		

### 3.2 Network structure of organ donation willingness, attitudes, and death attitudes

**Figure 1:**
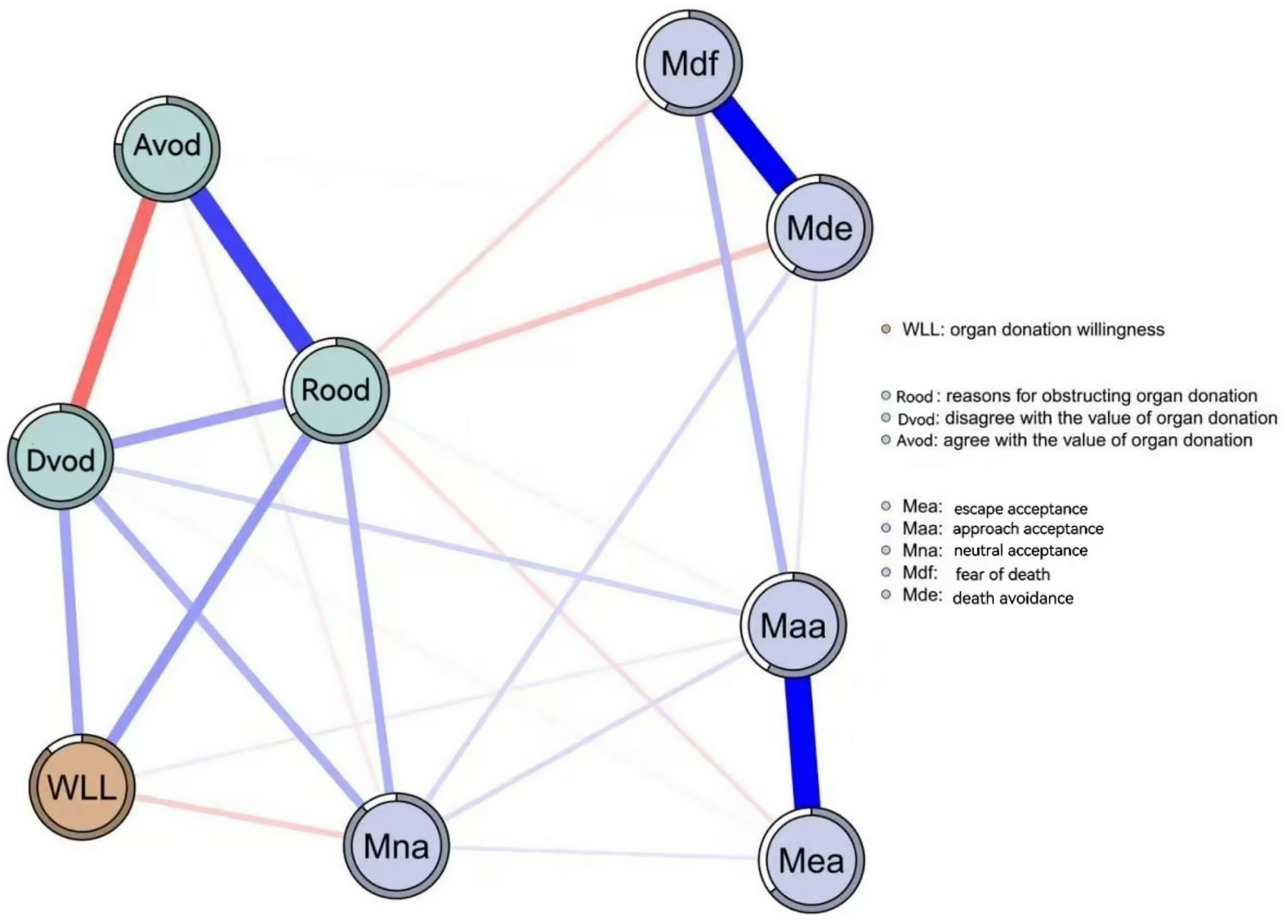
Network structure of organ donation willingness, donation attitudes, and death attitudes. Red edge: negative correlation; Blue edge: positive correlation. Edge thickness is proportional to the absolute edge weight magnitude (thicker lines indicate stronger conditional associations). The network layout was computed using the Fruchterman–Reingold algorithm. Only edges with non-zero partial correlations after LASSO regularization (*γ* = 0.5) are shown. WLL: organ donation willingness; Rood: reasons for obstructing organ donation; Dvod: disagree with the value of organ donation; Avod: agree with the value of organ donation; Mna: neutral acceptance; Mea: escape acceptance; Maa: approach acceptance; Mde: death avoidance; Mdf: fear of death.

Figure 1 presents the estimated network illustrating the interconnections among organ donation willingness, three dimensions of organ donation attitudes, reasons for obstructing organ donation (Rood), disagreement with the value of organ donation (Dvod), and agreement with the value of organ donation (Avod), and five dimensions of death attitudes: neutral acceptance (Mna), escape acceptance (Mea), approach acceptance (Maa), death avoidance (Mde), and fear of death (Mdf).

Among the death attitude dimensions, the strongest positive edge was observed between death avoidance (Mde) and fear of death (Mdf) (edge weight = 0.66), followed by the association between escape acceptance (Mea) and approach acceptance (Maa) (edge weight = 0.65).

Regarding organ donation-related variables, reasons for obstructing organ donation (Rood) and disagreement with the value of organ donation (Dvod) showed positive associations with donation willingness (WLL) (edge weight = 0.26, and 0.24, respectively).

#### 3.2.1 Centrality analysis

**Figure 2:**
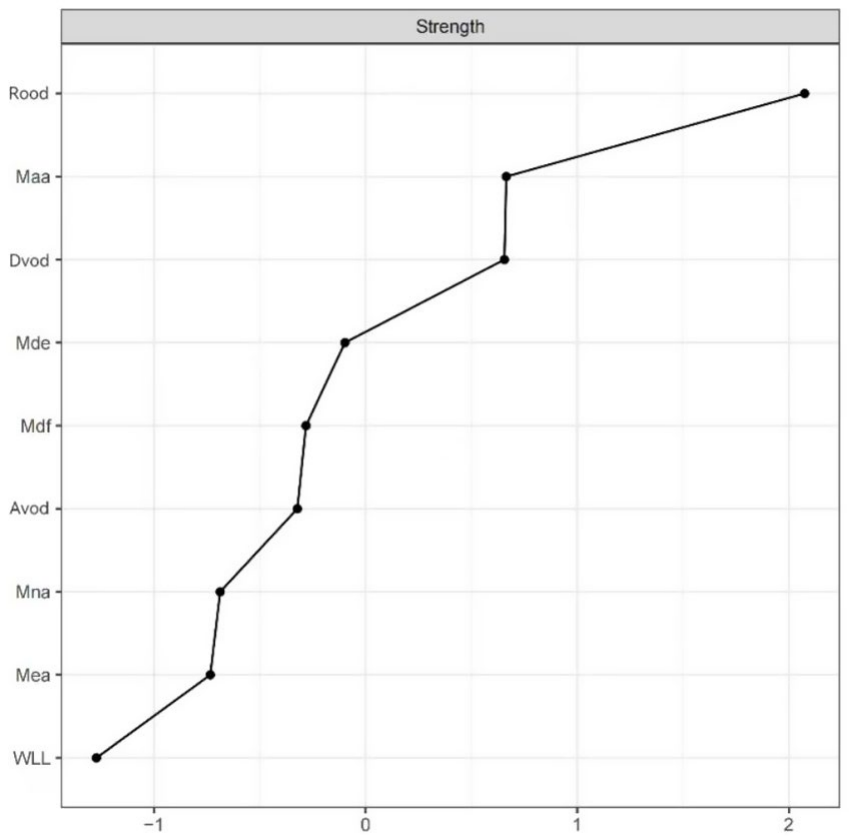
Standardized node strength centrality indices. Node strength (sum of absolute edge weights) is plotted as *z*-scores. Higher values indicate greater connectivity within the network. Error bars represent 95% bootstrap confidence intervals (1,000 samples). The CS coefficient for node strength was 0.62, indicating good stability. WLL: organ donation willingness; Rood: reasons for obstructing organ donation; Dvod: disagree with the value of organ donation; Avod: agree with the value of organ donation; Mna: neutral acceptance; Mea: escape acceptance; Maa: approach acceptance; Mde: death avoidance; Mdf: fear of death.

Figure 2 presents the standardized node strength centrality indices for all variables in the network. Reasons for obstructing organ donation (Rood) was identified as the most central node (strength = 2.07), followed by approach acceptance of death (Maa, strength = 0.66) and disagreement with the value of organ donation (Dvod, strength = 0.65).

Bootstrap resampling (1,000 iterations) indicated stable centrality estimates, with a correlation stability (CS) coefficient of 0.62 for node strength. The CS coefficient for edge weights was 0.75, indicating excellent stability.

#### 3.2.2 Bridge analysis

**Figure 3:**
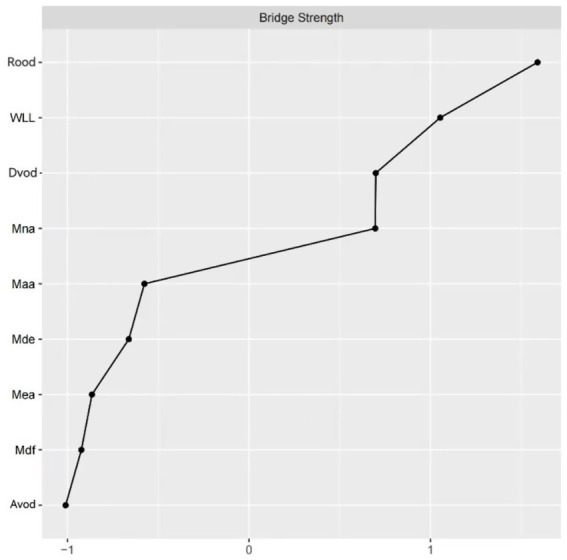
Standardized bridge strength centrality indices. Bridge strength quantifies the sum of edge weights connecting a node to nodes outside its own cluster. Clusters were defined as: donation attitude cluster (Rood, Avod, Dvod), death attitude cluster (Mna, Mea, Maa, Mde, Mdf), and willingness (WLL) as a separate cluster. Higher bridge strength indicates a node’s importance in linking different psychological domains. The CS coefficient for bridge strength was 0.75, indicating excellent stability. WLL: organ donation willingness; Rood: reasons for obstructing organ donation; Dvod: disagree with the value of organ donation; Avod: agree with the value of organ donation; Mna: neutral acceptance; Mea: escape acceptance; Maa: approach acceptance; Mde: death avoidance; Mdf: fear of death.

Figure 3 presents the standardized bridge centrality indices, identifying nodes that connect the donation attitude and death attitude clusters. Reasons for obstructing organ donation (Rood) was identified as the strongest bridge node (bridge strength = 1.59), followed by donation willingness (Willingness, bridge strength = 1.05). These nodes connected the donation attitude dimensions (e.g., Avod, Dvod) with the death attitude dimensions (e.g., Maa, Mdf). Bootstrap analysis (1,000 iterations) indicated stable bridge centrality estimates, with a CS coefficient of 0.75 for bridge strength.

## 4 Discussion

This study examined Chinese university students’ attitudes toward organ donation by focusing on three key psychological constructs: donation willingness, donation attitudes, and death attitudes. Network analysis was employed to construct a graphical model encompassing three dimensions of organ donation attitudes, five dimensions of death attitudes, and donation willingness. The analysis identified central nodes within this interconnected structure, providing a basis for targeted intervention strategies.

Most respondents were at the stage of “having considered but not yet decided” regarding organ donation. This finding is consistent with Chen et al. (24), who reported that although Chinese university students generally possess basic awareness of organ donation, their understanding remains superficial, potentially limiting deeper reflection and actual commitment to donation.

The network analysis revealed a positive association between reasons for obstructing organ donation and disagreement with the value of organ donation. This association may reflect that individuals who experience discomfort with surgical procedures tend to develop reservations about the value of organ transplantation (30). Such apprehensions may manifest as beliefs that one’s body is unsuitable for donation or as ethical concerns regarding the potential misuse of donated organs (e.g., allocation to undeserving recipients). Conversely, individuals who endorse organ donation tend to perceive greater value in donating viable organs to others after death.

In addition, no significant differences in donation willingness were observed across gender, place of residence, or religious affiliation. This finding may be related to the relatively homogeneous educational background of the sample. Psychological factors may play a more prominent role in shaping perceptions and willingness toward organ donation in this population.

Among the five dimensions of death attitudes, death avoidance was positively associated with fear of death, and approach acceptance was positively associated with escape acceptance. In contrast, neutral acceptance showed minimal associations with the other four dimensions. Although both approach acceptance and escape acceptance reflect positive orientations toward death, they represent subjective tendencies that differ from the neutral stance of neutral acceptance. Individuals endorsing either approach acceptance or escape acceptance acknowledge death; however, their acceptance is subjectively motivated, by fear in escape acceptance or by a proactive embrace of mortality in approach acceptance (31).

Centrality analysis indicated that reasons for obstructing organ donation and disagreement with the value of organ donation were the most central nodes within the organ donation attitude network. Consistent with the KAP model, these nodes also showed the strongest associations with donation willingness, supporting the model (9). Both supportive and opposing attitudes were associated with differences in donation willingness, consistent with findings by Fan et al. (7). These findings suggest that strengthening public awareness may be a promising direction for future intervention research (32). Universities may consider targeted educational initiatives (e.g., lectures, workshops, or awareness campaigns) to improve students’ understanding of organ donation (33–35).

Although willingness is conceptualized as an outcome variable, its high bridge strength (1.05) suggests that it may also be closely linked to multiple attitudinal dimensions. For example, higher willingness may be associated with a greater likelihood of initiating family discussions about organ donation. Family discussions have been associated with increased likelihood of family consent to donation (20, 21), and are particularly important in the Chinese cultural context for addressing concerns about deceased organ donation (7). These findings suggest a potential feedback loop, in which higher willingness may be associated with increased family communication, potentially reducing perceived barriers (Rood) or reinforcing positive attitudes (Avod). Future longitudinal or experimental studies are needed to test this potential bidirectional relationship.

The network analysis identified disagreement with the value of organ donation as the third most central node within the psychological network, suggesting its important role in organ donation willingness among Chinese university students. Given its high nodal strength, this construct likely acts as a leverage point in the network, implying that interventions aimed at reframing the moral or social value of organ donation could substantially enhance students’ willingness by weakening the negative influence of this central node.

Approach acceptance, characterized by a proactive embrace of mortality (e.g., viewing death as a gateway to an altruistic legacy), was positively associated with donation willingness in this study. The bridge function of approach acceptance suggests that it may link positive death attitudes with donation-related decision-making.

From a cultural perspective, Confucian values emphasizing the virtue of leaving a moral legacy may contribute to the centrality of approach acceptance in this sample; however, this interpretation remains speculative without direct measurement of cultural orientation (36). Future research should incorporate validated measures of cultural values to test this hypothesis. Traditional death taboos may contribute to ambivalence, which could be addressed through interventions that reframe organ donation as an act of moral and cultural continuity.

Although immediate donors are typically older adults, the present findings from a student sample have implications for broader populations. The identified central nodes, particularly perceived barriers to donation and approach acceptance of death, are psychological mechanisms likely operative across age groups. Under China family consent requirement, students also occupy a unique position to disseminate donation attitudes to older relatives through family discussions. Thus, defining students as a critical population for promotion is justified not by their role as immediate donors, but by their function as attitudinal transmitters and a strategic starting point for intervention development that may subsequently extend to older adults.

### 4.1 Limitations

Several limitations should be acknowledged. First, the cross-sectional design precludes causal inference. Network analysis identifies structural relationships but does not establish whether modifying one node causally affects another. Centrality should not be equated with intervention efficacy; rather, the findings identify candidate targets for future longitudinal or experimental studies. Second, sampling limitations restrict generalizability. The sample consisted exclusively of university students from four institutions (two in eastern China and one in southern China), and the overrepresentation of male students (63.4%) may limit representativeness. The findings may not generalize to older adults, less educated individuals, or rural populations, where traditional beliefs may differ. Nevertheless, whether the identified network structure generalizes to older adults remains to be tested in future research. Furthermore, although we achieved a high completion rate (96.0%) among those who started the survey, we could not calculate the initial response rate. Third, measurement limitations include reliance on self-report, the absence of objective behavioral outcomes (e.g., actual donor registration). Furthermore, donation willingness was assessed using a single item, which may not fully capture the multidimensional nature of behavioral intention and may be more susceptible to measurement error than multi-item scales. Although Harman’s single-factor test suggested no severe common method bias, it is insufficient; future studies should incorporate marker variables or longitudinal designs. Fourth, psychometric considerations: although the organ donation attitude scale has been widely used in Chinese populations (25), confirmatory factor analysis (CFA) was not conducted to verify its factor structure in this sample. This limitation should be addressed in future research. Fifth, network analysis limitations: although the CS coefficients for node strength (0.62) and bridge strength (0.75) indicate good stability, bridge centrality estimates in smaller networks may be less precise. The results should be interpreted as exploratory. Additionally, the interpretation linking approach acceptance to Confucian values remains speculative without direct measurement. Finally, the specific cultural context (Chinese university students) may limit generalizability to other populations or countries with different organ donation systems or cultural attitudes toward death.

## Data Availability

The raw data supporting the conclusions of this article will be made available by the authors, without undue reservation.
